# Spectroscopic Signature of Red Blood Cells in a D-Galactose-Induced Accelerated Aging Model

**DOI:** 10.3390/ijms22052660

**Published:** 2021-03-06

**Authors:** Aneta Blat, Tetiana Stepanenko, Katarzyna Bulat, Aleksandra Wajda, Jakub Dybas, Tasnim Mohaissen, Fatih Celal Alcicek, Ewa Szczesny-Malysiak, Kamilla Malek, Andrzej Fedorowicz, Katarzyna M. Marzec

**Affiliations:** 1 Jagiellonian Center for Experimental Therapeutics, Jagiellonian University, 14 Bobrzynskiego Str., 30-348 Krakow, Poland; aneta.blat@doctoral.uj.edu.pl (A.B.); tetiana.stepanenko@student.uj.edu.pl (T.S.); katarzyna.bulat@jcet.eu (K.B.); olawajda@agh.edu.pl (A.W.); jakub.dybas@jcet.eu (J.D.); tasnim.mohaissen@jcet.eu (T.M.); f.celal.alcicek@jcet.eu (F.C.A.); ewa.szczesny@jcet.eu (E.S.-M.); 2 Faculty of Chemistry, Jagiellonian University, 2 Gronostajowa Str., 30-387 Krakow, Poland; kamilla.malek@uj.edu.pl; 3 Faculty of Materials Science and Ceramics, AGH University of Science and Technology, Al. Mickiewicza 30, 30-059 Krakow, Poland; 4 Faculty of Pharmacy, Jagiellonian University Medical College, 9 Medyczna St., 30-688 Krakow, Poland; 5 Chair of Pharmacology, Jagiellonian University Medical College, 16 Grzegorzecka Str., 31-531 Krakow, Poland; andrzej.fedorowicz@uj.edu.pl

**Keywords:** red blood cells (RBCs), aging, RBC membranes, D-galactose-induced accelerated aging mouse model, vibrational spectroscopy, Raman spectroscopy, Fourier transform infrared spectroscopy–attenuated total reflectance (FTIR–ATR)

## Abstract

This work presents a semi-quantitative spectroscopic approach, including FTIR–ATR and Raman spectroscopies, for the biochemical analysis of red blood cells (RBCs) supported by the biochemical, morphological and rheological reference techniques. This multi-modal approach provided the description of the RBC alterations at the molecular level in a model of accelerated aging induced by administration of D-galactose (D-gal), in comparison to natural aging. Such an approach allowed to conclude that most age-related biochemical RBC membrane changes (a decrease in lipid unsaturation and the level of phospholipids, or an increase in acyl chain shortening) as well as alterations in the morphological parameters and RBC deformability are well reflected in the D-gal model of accelerated aging. Similarly, as in natural aging, a decrease in LDL level in blood plasma and no changes in the fraction of glucose, creatinine, total cholesterol, HDL, iron, or triglycerides were observed during the course of accelerated aging. Contrary to natural aging, the D-gal model led to an increase in cholesterol esters and the fraction of total esterified lipids in RBC membranes, and evoked significant changes in the secondary structure of the membrane proteins. Moreover, a significant decrease in the phosphorous level of blood plasma was specific for the D-gal model. On the other hand, natural aging induced stronger changes in the secondary structures of the proteins of the RBCs’ interior. This work proves that research on the aging mechanism, especially in circulation-related diseases, should employ the D-gal model with caution. Nonetheless, the D-gal model enables to imitate age-related rheological alterations in RBCs, although they are partially derived from different changes observed in the RBC membrane at the molecular level.

## 1. Introduction

Over time, each cell loses its effectiveness for defending itself from reactive oxygen species (ROS), reactive nitrogen species (RNS), and other free radical species, which leads to progressive cellular oxidation [1]. On the other hand, it is also known that oxidation not only accelerates the development of many diseases, but also may initiate disease onset. Aging-related processes have an impact on each living cell and can be linked to most of the diseases of affluence. Blood plays a special role in the aging mechanism, as it is not only a supplier of oxygen and nutrients for tissues, but it also removes metabolic waste and oxidative species. Therefore, during oxidative stress, all blood cells are exposed to an environment rich in ROS, NOS, and free radical species. As was previously reported, red blood cells (RBCs) exhibit time-dependent damage through characteristic changes in the size and shape of their cell morphology, macrovesicle production, and changes in the biochemical structure of their cytosol and membranes [2]. It was previously reported that aging influences the unsaturated fatty acyl side chains and phospholipid fraction in the RBC membrane, which is manifested by a decrease in phospholipids and unsaturation levels [3]. In healthy mammalian RBC membranes, choline-containing phospholipids and sphingomyelin are mainly found in the outer monolayer, while the amino phospholipids are predominantly or exclusively found in the inner monolayer, but apart from phospholipids, the erythrocyte membrane also contains cholesterol and glycosphingolipids [4]. It is known that an increase in the cholesterol-to-phospholipid ratio from 1.28 to 2.0 in the RBC membrane results in a decrease in its permeability [5]. The structure and properties of the RBC membrane are not only defined by membrane lipids, but also by lipid-protein interactions [6]. The aging process also has an impact on the bicarbonate/chloride exchanger 1 (AE1, Band 3) in the RBC membrane, which is involved in gas exchange and functions as a major site of attachment of the cytoskeleton to the erythrocyte membrane [7,8]. Recently, it was also reported that prolonged storage of packed red blood cells results in in the occurrence of irreversible changes in both secondary and quaternary structures of Hb, with subsequent impairment in the T⇿R transition [9]. Moreover, in high-stress conditions, hemoglobin in the RBCs can be degraded by peroxides to heme and free iron, which is released out of the cell. It was reported that oxidized, senescent, or stored RBCs are accompanied by RBC-derived vesicles [10]. RBCs lose approximately 20% of their volume during their life through the emission of vesicles [11]. Vesiculation is a way to get rid of dangerous molecules, such as oxidized proteins or denatured hemoglobin [12]. Multilevel changes in the lipids and proteins in RBC membranes, caused by aging, cause an increase in RBC deformability [6] and an unfavorable change in blood flow, which promotes additional oxidative stress [13], proneness to atherosclerotic lesions, and an increase in blood viscosity [14].

In this work we provide a description of the biochemical, mechanical, and functional changes in RBCs in a mouse model of accelerated aging induced by D-galactose. If this model successfully mimics the state of RBCs in natural aging, its application will allow to shorten a study’s duration and secure a higher animal survival rate, in comparison with natural aging [15,16]. Consumption of D-galactose within the limits of the recommended daily dose does not pose any health threat, because it is metabolized and excreted. In contrast, the excess of this sugar leads to the production of reactive oxygen species, when galactose oxidase converts it into aldose and hydrogen peroxide. Increased level of reactive oxygen species results in oxidative stress, inflammation, mitochondrial dysfunction, and apoptosis [17,18,19]. It was previously reported that administration of a low dose of D-galactose to mice causes neurological disorders [20], a shortened lifespan [16], decreased neuromuscular activity, increased production of free radicals [20], neurodegeneration [21,22,23], cognitive impairment [21,23,24], decreased immune response [16,25], decreased reproductive capacity [26], and decreased neurogenesis, which may be related to an increase in the level of reactive oxygen radicals (ROS) [27]. Since these features resemble phenomena occurring during the aging process, the model of D-galactose-induced aging was previously used in studies of the mechanism of aging and drug testing [28]. Features proving the usefulness of this model include the accumulation of harmful metabolites inside the cells, resulting from a decrease in lysosomal activity and macro-autophagy [29,30] as well as changes in the content of antioxidant enzymes in the brain, heart, and liver [20]; reduction in the level of superoxide dismutase (T-SOD) [20,31] and glutathione peroxidase (GSH-PX) [31]; a decrease in the lymphocyte proliferation rate and in the production of IL-2 [32,33]; and an increase in infectious diseases, malignancies, and autoimmune diseases resulting from age [34]. Various theories have been proposed to explain the mechanism of D-galactose-induced changes, pointing either to the accumulation of galactothiol [20], resulting in mitochondrial dysfunction [35], or to the accumulation of the advanced glycation end-products (AGEs) [25,36]. AGEs are involved in the development of pathologies, such as diabetes, atherosclerosis, nephropathy, infection, Alzheimer’s disease, cataracts, arthritis, and oxidative damage in various organs [28,37,38,39]. The third theory assumes that an increased level of D-galactose causes an increase in the level of hydrogen peroxide formed during D-galactose metabolism, and a decrease in superoxide dismutase (SOD) content and redox homeostasis disorders [20].

In this work, we studied alterations in RBCs in 5-month-old male C57BL/6J mice subdued to accelerated aging by administration of D-galactose. Obtained results were compared to alterations observed in the RBCs of intact 5-month-old and 7-month-old male C57BL/6J mice, in order to define which age-related biochemical RBC membrane changes and alterations in morphology parameters and RBC deformability are well reflected in the applied model of accelerated aging. Such an approach was necessary to define whether the model of D-galactose-induced accelerated aging can be applied in studies focused on the aging mechanisms in circulation-related diseases.

## 2. Results

### 2.1. Rheological and Morphological RBC Analysis

**Figure 1:**
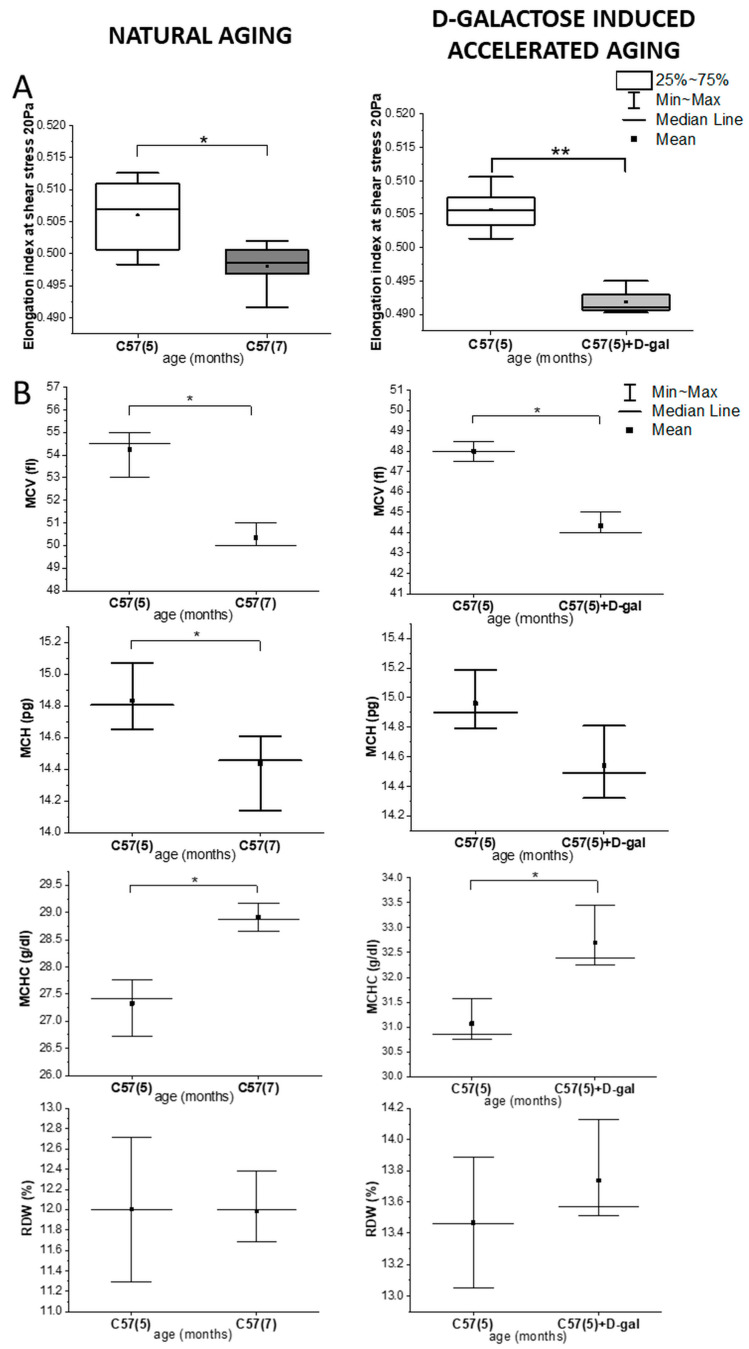
Rheological and morphological changes in the red blood cells (RBCs) in natural aging and the D-galactose (D-gal) model. (**A**) Changes in the elongation index at a shear stress of 20 Pa; and (**B**) the red blood cell indices, mean corpuscular volume (MCV), mean corpuscular hemoglobin (MCH), mean corpuscular hemoglobin concentration (MCHC), red cell distribution width (RDW), in naturally aging C57BL/6J mice (5- and 7-month-old male C57BL/6J mice, *n* = 4 and *n* = 3, respectively) and the D-galactose-induced accelerated aging mouse model (5 month-old C57BL/6J male mice, *n* = 3) with the control group (5 month-old C57BL/6J male mice, *n* = 3). Data distribution is presented as box plots (median and interquartile range, min–max whiskers) and interval plot (mean value, median, min-max whiskers). Statistical significance of the obtained values was tested with a Mann–Whitney test (* *p* < 0.05; ** *p* < 0.01).

Figure S1 summarizes all analyzed morphological parameters, while Figure 1 presents those that were previously defined as having prognostic value in relation to variations of RBC deformability observed in natural aging and D-gal-induced accelerated aging [40]. According to earlier studies, mean values of the morphological parameters of RBCs vary, depending not only on C57BL/6J mice age and sex, but also on the breeding [41,42,43]; therefore, comparisons presented in this work were shown always for animals of the male sex and from the same breeding. A statistically significant decrease in RBC deformability in the D-gal model reflected the same trend observed in natural aging (Figure 1A). Like the RBCs from naturally aging mice, the RBCs from the D-gal model showed a statistically significant decrease (*p* < 0.05) in the mean value of MCV (Figure 1B) and an increase (*p* < 0.05) in the mean value of MCHC (Figure 1B). Although a decrease observed in the mean value of the MCH parameter in the D-gal model is not significant, the trend is the same as in natural aging. Further on, no significant changes were observed in RDW, HGB, or HCT (Figure 1B and Figure S1) in the D-gal model; again, similarly as in natural aging [3].

### 2.2. Biochemical Blood Plasma Analysis

**Figure 2:**
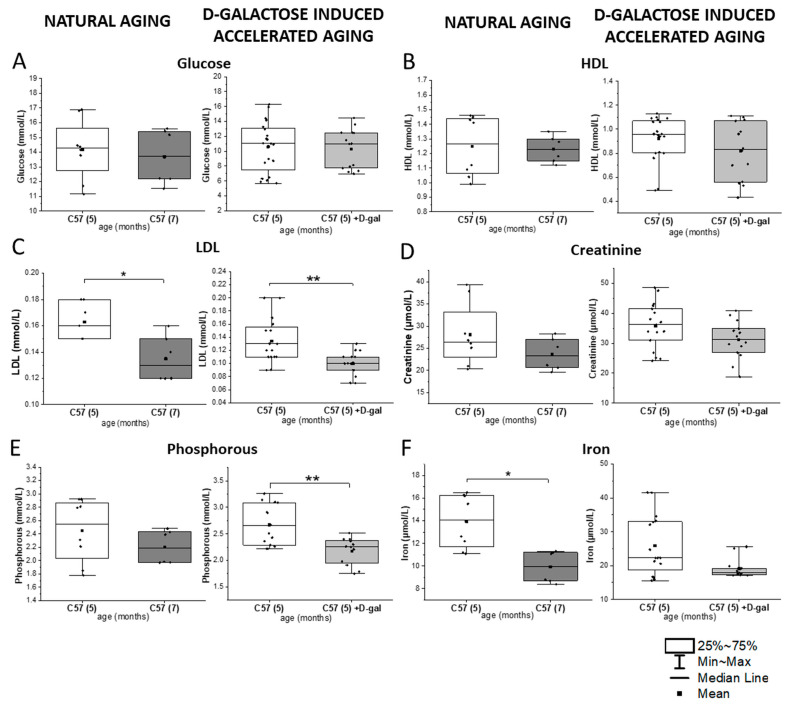
Biochemical parameters in the blood plasma in natural aging and the D-gal model. (**A**) Glucose, (**B**) HDL, (**C**) LDL, (**D**) creatinine, (**E**) phosphorous, and (**F**) iron levels in 5- and 7-month-old male C57BL/6J mice (*n* = 4 and *n* = 3, respectively) in natural aging and 5-month-old male C57BL/6J mice in the D-galactose-induced aging model (*n*=7) compared with the control group (*n* = 10). The data distribution is presented as boxes (median and interquartile range, min-max whiskers). The statistical significance of the obtained values was tested with a Mann–Whitney test (* *p* < 0.05; ** *p* < 0.01).

As presented in Figure 2 and Figure S2, the D-gal model induced some changes in the blood plasma, reflecting those observed during natural aging, such as a significant decrease in the LDL level. Similarly, as in natural aging, no changes were observed in the fraction of glucose, creatinine, total cholesterol, HDL, LDH, iron, or triglycerides in the induced accelerated aging. Only a significant decrease in phosphorous was specific for the D-gal model.

### 2.3. Spectroscopic Analysis of the RBC Membranes

**Figure 3:**
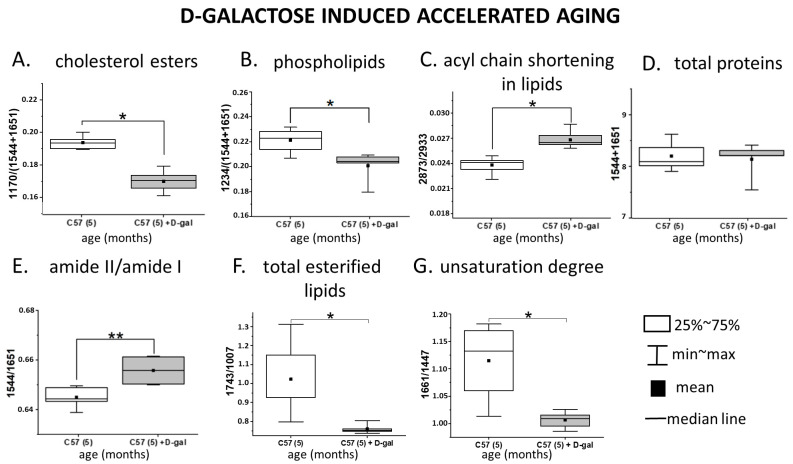
Spectroscopically derived biochemical profile of the RBC membranes in natural aging and the D-gal model. Ratios calculated for the integral absorbances of the ATR–FTIR spectra (**A**–**E**) and integral intensities of the Raman spectra (**F**,**G**), showing alterations in the biochemical composition of the RBC membranes due to the D-galactose-induced accelerated aging (*n* = 3, 5-month-old D-galactose-fed C57BL/6J male mice) in comparison to control mice (*n* = 3, 5-month-old C57BL/6J male mice). Integration regions for IR bands: amide I—1651 cm^−1^ (1687–1605 cm^−1^); amide II—1544 cm^−1^ (1560–1502 cm^−1^); CH_2_ symmetric stretch—2933 cm^−1^ (2863–2847 cm^−1^); CH_3_ asymmetric stretch—2873 cm^−1^ (2965–2936 cm^−1^); PO_2_^–^ asymmetric stretch—1236 cm^−1^ (1261–1214 cm^−1^); –CO–O–C stretch—1167 cm^−1^ (1191–1144 cm^−1^); amide I—1651 cm^−1^ (1687–1605 cm^−1^); amide II—1544 cm^−1^ (1560–1502 cm^−1^). Integration regions for the Raman bands: symmetric stretch of C=O—1743 cm^−1^ (1715–1764 cm^−1^) symmetric stretch C=C—1661 cm^−1^ (1655–1670 cm^−1^), scissoring and bending vibrations of CH_2_ and CH_3_—1447 cm^−1^ (1420–1480 cm^−1^), aromatic ring breathing mode–phenylalanine—1007 cm^−1^ (990–1015 cm^−1^). Changes in the RBC membranes in natural aging are presented in Figure S4. Normality was assessed using the Shapiro–Wilk test. The data are expressed as box plots (median and interquartile range) and the significance was calculated using the Mann–Whitney test (* *p* < 0.05, ** *p* < 0.01).

Isolated RBC membranes derived from the control and D-gal mice were examined with use of FTIR-ATR and RS (488 nm laser excitation). Each technique focuses on various physical effects, being sensitive to different type of vibrations, delivering synergetic and mutually supporting information. Averaged FTIR–ATR and RS spectra collected from isolated membranes are presented in Figure S3. Differences between spectroscopic features of the RBC membrane samples from the D-gal model and control (Figure 3), as well as natural aging (Figure S4), are presented in a semi-quantitative manner as box charts representing the ratios of the integral intensities of the chosen marker bands. A comparison between the results presented in Figure 3 and S4 shows that changes in the main components of the RBC membranes, such as the fractions of phospholipids, lipid unsaturation, and acyl chain shortening, exhibit the same trends in the D-gal model as in natural aging. The total protein content in the isolated membranes of the control and D-gal mice is presented as the sum of the amide I and II integral intensities [44] and remained unchanged in D-gal compared to the control (Figure 3D), similarly to natural aging (Figure S4D) [3,39]. A statistically significant decrease induced by D-gal was observed for absorbance at 1236 cm^−1^, assigned to the asymmetric stretching modes of the PO_2_^−^ groups, indicating a decrease in the phospholipid content (Figure 3B) and staying in accordance with Figure S4B, presenting the process of natural aging. Additionally, RS spectra revealed a decrease in the lipid unsaturation level expressed by the ratio of the 1661 cm^−1^ band, corresponding to the C=C stretching vibrations, to the 1447 cm^−1^ band attributed to the scissoring mode of the CH_2_/CH_3_ groups (Figure 3G). This change was found in concordance with a statistically significant increase in the FTIR band ratio of the 2873 and 2933 cm^−1^ bands (the CH_3_ symmetric stretching and CH_2_ asymmetric stretching, respectively), indicating acyl chain shortening in lipids due to lipid peroxidation [45,46] (Figure 3C). Contribution of proteins to these bands is constant, as the sum of amide I and II bands indicated. The process of lipid oxidation was similar in natural aging (Figure S4C,G).

D-gal also induced a change in the ratio of amide II to amide I in FTIR spectra of RBC membranes (Figure 3E), contrary to typical aging (Figure S4E). Moreover, a statistically significant decrease in absorbance of the FTIR band at 1171 cm^−1^, assigned to the –CO-O-C- asymmetric stretching vibrations of cholesterol esters (Figure 3A), was observed only in the D-gal model [47]. A similar change appeared in the total content of the esterified lipids, expressed in the RS spectra by the ratio of 1743 and 1007 cm^−1^ bands, assigned to the C-O stretching and phenylalanine modes, respectively (Figure 3F) [48]. Interestingly, an opposite trend in changes in the cholesterol esters’ content and total esterified lipids was observed in natural aging (Figure S4A). A downward trend in the integral intensity of the band at 1738 cm^−1^ (derived from stretching modes of the ester C=O group), contributing to the esterified lipids and triglycerides, was also found only in the D-gal model (Figure S5A) [47].

### 2.4. Spectroscopic Analysis of Intact RBCs—Insight into Cytosol Alteration

**Figure 4:**
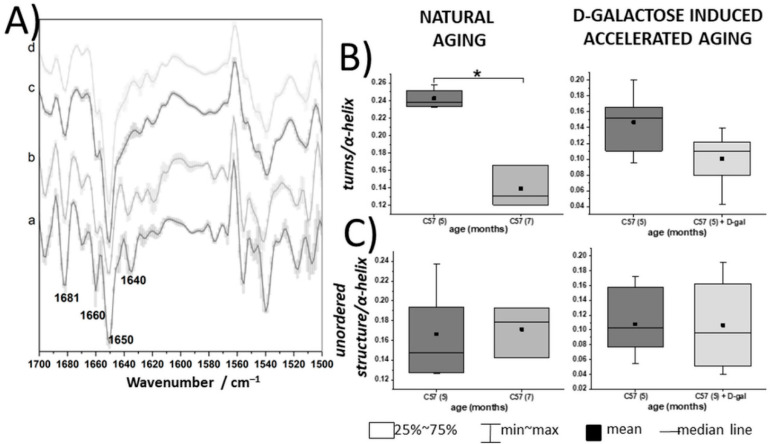
Spectroscopically derived biochemical profile of intact RBCs in natural aging and the D-gal model. (**A**) Second derivative FTIR–ATR spectra (with SD) of unfixed, intact RBCs taken from 5- and 7-month-old C57BL/6J mice (a and b, respectively) (*n* = 3–4) and 5-month-old C57BL/6J mice (*n* = 3) and treated with D-galactose (c and d, respectively) (*n* = 3), displayed in the 1700–1500 cm^−1^ spectral region; (**B**,**C**) ratios of the integral absorbances at 1660/1650 and 1640/1650 cm^−1^, respectively, comparing natural aging and a model of D-galactose-induced accelerated aging, in terms of alterations in the secondary structures of the RBC proteins, i.e., turns to α-helices and unordered conformations to α-helices, respectively. Normality was assessed using the Shapiro–Wilk test. The data are expressed as box plots (median and interquartile range) and the significance was calculated using a Mann–Whitney test (* *p* < 0.05).

Intact erythrocytes were investigated only with use of the ATR–FTIR technique, due to the resonance Raman scattering of the heme proteins, which prevents accessing other information than those related to the Hb molecules. [49]. The second derivative of the averaged ATR–FTIR spectra with standard deviation (SD), collected from the intact RBCs, are presented in Figure 4A, while box charts representing the ratios of the integral intensities of the chosen marker bands for intact RBCs are presented in Figure 4B,C. Total protein content of the control and D-gal RBCs was presented as a sum of the amide I and II integral intensities [44] and remained unchanged in the D-gal model compared to the control animals, also in case of intact erythrocytes (Figure S6A). The second derivative FTIR spectra revealed a downward trend in the ratio of turns to α-helices in the intact RBCs from the D-gal model, expressed by the 1660/1650 cm^−1^ ratio, which was statistically significant in natural aging (Figure 4A,B) [3]. Similarly, as in natural aging, no changes were seen in the ratio of unordered proteins structure to α-helices.

## 3. Discussion

Some alterations in the RBCs induced by the D-gal model are also characteristic for natural aging. This model causes a significant decrease in RBC deformability, which affects their rheological properties and their ability to squeeze through microvessels. When compared with the control, deterioration of RBC morphology in the D-gal model follows the trend observed in natural aging of C57BL/6J mice, namely, a decrease in MCV and an increase in MCHC. It is worth highlighting that comparison of the dry smears of fresh RBCs isolated from the studied groups of animals did not provide any statistically significant changes in their RBC shape (majority with a bio-concave shape). This stays in agreement with our previous results, where we have evaluated the morphological alterations and ultrastructural features among the control and natural aging groups in C57BL/6J RBCs using not only the classical dry smear analysis, but also nano-scale AFM to finely analyze the RBC ultrastructural features [3]. Those results clearly proved that only detailed AFM analysis provided insight into an age-dependent decrease in RBC height and no signs of ultrastructural deteriorations in RBC morphology were found.

The biochemical composition of RBC membranes observed in the D-gal model and natural aging includes a decrease in the phospholipid amount, lipid unsaturation level, and an increase in acyl chain shortening. Even though some alterations are not statistically significant in the D-gal model, their direction remains the same as in natural aging. Here we may list the changes to the secondary structure of the proteins of intact RBCs, revealed by a decrease in the ratio of turns to α-helices, a decrease in MCH and LDL, and lack of changes in RDW and blood plasma parameters, such as HDL, cholesterol, triglycerides, and LDH. Altogether, these suggest that the D-gal model mimics both the mechanical and functional properties of the RBCs of naturally aging mice.

It was previously reported that alterations in the secondary structures of proteins in intact RBCs may be regarded as biomarkers of aging or specific diseases. Progressive formation of β-sheets accompanied by a decrease in α-helices was observed in aging of stored human RBCs [9], while a significant increase in the ratio of unordered conformations to α-helical structures was characteristic for RBCs in the due course of atherosclerosis progression. Moreover, changes in the ratio of turns to α-helices were more specific for aging processes in mice [3]. Changes to the secondary structures of proteins in the intact RBCs derived from D-gal-treated mice resemble changes seen during natural aging of C57BL/6J mice. A reduced ratio of turns to α-helices suggests aggregation of proteins, which can be stimulated, promoted, and accelerated by advanced glycation, i.e., formation of advanced glycation end-products (glycated proteins or lipids) during d-galactose transformations [25,50,51]. This process is suggested to be the main mechanism of D-gal-induced aging, and is also associated with an increase in oxidative stress [25,52]. Protein glycation occurring in RBCs during D-galactose-induced accelerated aging was also confirmed by biochemical changes in plasma, where a statistically significant decrease in the glucose concentration was observed (the drop is probably caused by the reaction of the sugar with amine groups of the RBC proteins). Comparisons of the alterations of the lipid profile of the RBC membranes shows a significant decrease in phospholipids and lipid unsaturation in both natural aging as well as the D-gal model. A decrease in the level of phospholipids causes RBC membrane remodeling, which results from the lipid peroxidation caused by oxidative stress [3,44]. Products of this process induce a gradual impairment of the membrane, which activates ion channels, resulting in calcium ion influx. In turn, the presence of Ca^2+^ causes activation of phospholipase and a subsequent cleaving of the phospholipids. Peroxidation in the RBCs is also manifested by the shortening of the fatty acid chains, which is expressed by an increase in the ratio of the CH_3_/CH_2_ stretching vibrations [46,53]. Like in natural aging, a significant decrease in the LDL level in blood plasma and no changes in the fraction of glucose, creatinine, total cholesterol, HDL, iron, or triglycerides were observed in the D-gal model. These results clearly suggest some similarities between the degradation mechanisms observed in the RBCs from the D-gal-treated animals and those that aged naturally.

On the other hand, a detailed analysis of the biochemical profile of the blood plasma and RBC membranes shows some differences between the D-gal model and natural aging. These differences indicate some variations in the nature of the RBC alterations at the molecular level. Significant, specific changes in blood plasma of the D-gal model refer to a decrease in phosphorous fraction, which can be related to the galactose metabolic pathway that includes phosphorylation of D-galactose to galactose-1-phosphate (gal-1-P) via the galactokinase enzyme [54,55]. Enzyme deficiency in this pathway results in a disease called galactosemia [54,55,56]. Previous studies in humans showed that phosphorus in the RBCs and plasma may be decreased due to accumulation of gal-1-P in the cells [57,58,59,60]. In the D-gal model, a high level of galactose phosphorylation may be the cause of a decrease in the phosphorus fraction. Our results also agree with Machado et al., who showed that accumulation of gal-1-P causes a decrease in the intracellular phosphate levels in yeast models of galactosemia [61]. We discovered a decrease in both cholesterol esters and total esterified lipids in the RBC membranes in the D-gal mouse model, which is contradictory to their increase in RBC membranes in naturally aging mice. Moreover, contrary to natural aging, D-gal caused significant changes in the secondary structures of the membrane proteins. On the other hand, natural aging induced stronger changes in the secondary structures of the RBCs’ interior than the D-gal model. This divergence may indicate slightly different mechanisms of RBC degradation in the studied D-gal aging model than the natural aging process.

## 4. Materials and Methods

### 4.1. Animal Models and Blood Collection

All experiments were carried out in accordance with the Guidelines for Animal Care and Treatment of the European Union and with consent issued by the First Local Ethical Committee on Animal Testing at the Jagiellonian University in Krakow (Resolution No. 72/2017 of 28.06.2017 and Resolution No. 262B/2019 of 24.04.2019).

**Figure 5:**
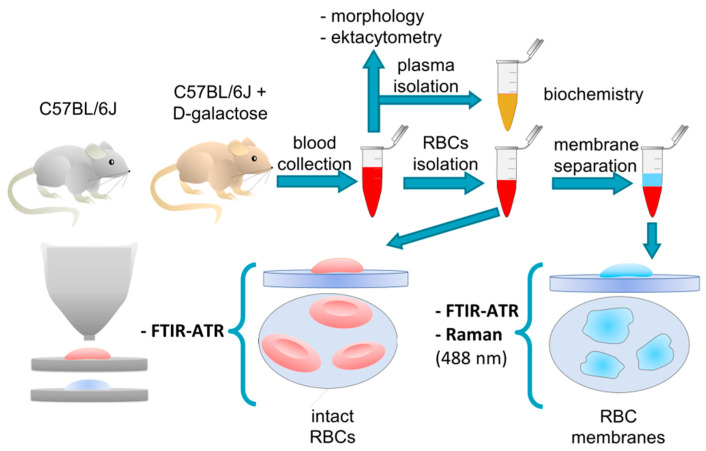
A schematic of the performed experiment.

The scheme of the experiment based on a mouse model of D-galactose-induced aging (D-gal model) is presented in Figure 5**.** The 5-month-old male C57BL/6J mice were either left intact, thus making a control group (*n* = 3–10), or treated intraperitoneally with 50 mg of D-galactose per kg of body weight, daily for 8 weeks (*n* = 3–7). To compare the aging model with natural aging in mice, additional experiments with the use of 5- and 7-month-old male C57BL/6J mice (*n* = 4 and *n* = 3, respectively) were conducted. However, all animals were housed 2–3 per cage in a temperature-controlled environment (22–25 °C) with a twelve-hour cycle of light/dark and unlimited access to food and water; also, the mice used in natural aging and the D-gal-induced model were acquired from different breeding stocks from various labs. Therefore, direct comparison of the naturally aged and D-gal-treated model animals could not be performed, and the data was always referred to appropriate breed-match controls. Mice were anesthetized with an intraperitoneal overdose of 100 mg/kg ketamine and 10 mg/kg xylazine. The chest was cut open and blood samples were drawn from the right heart ventricle into a 10 units/µL heparin syringe.

### 4.2. RBCs Isolation and Membrane Separation

RBCs were gravity-isolated from the whole volume of collected blood by a 15-min centrifugation (Sigma Laborzentrifugen GmbH, Osterode am Harz, Germany) in room temperature with 800× *g* acceleration and soft braking. Afterwards, the plasma and remaining coat containing platelets and white blood cells were removed. RBCs were washed with a Ringer-Tris buffer solution (Sigma-Aldrich, Saint-Louis, MI, USA) containing NaCl, CaCl_2_, KCl, MgSO_4_, Tris Base, glucose, and bovine serum albumin, and with the pH adjusted to 7.35–7.45 [40]. Sample centrifugation was repeated twice (800× *g*, 10 min, RT, soft braking). Before each centrifugation, Ringer-Tris buffer solution was added and afterwards the supernatant was discarded. The purity of the isolated RBCs was verified by measuring the blood count and determining the content of white blood cells in the sample (it should not exceed 200/µL).

As shown in Figure 5, a part of the isolated RBCs was left in the Ringer-Tris buffer solution (Hct = 10%) for FTIR–ATR (Fourier transform infrared spectroscopy–attenuated total reflection) studies. The remaining part was used for membrane isolation. The samples from the control and aged group were pooled into groups of 3 and frozen overnight in a 0.9% NaCl aqueous solution (Hct = 10%). The NaCl crystallization caused mechanical damage to the RBC membranes, leading to hemoglobin release. After defrosting, the samples were centrifuged for 10 min in 4 °C with 3000× *g* acceleration and soft braking. Next, the RBC membranes were aspirated from the upper layer (above the hemoglobin layer), suspended in a 0.9% NaCl aqueous solution, and prepared for FTIR–ATR and Raman studies.

### 4.3. Rheological, Morphological, and Biochemical Analysis

After collection, the whole blood samples were immediately subjected to RBC deformability tests and complete blood count analysis. RBC deformability was assessed by a slit-flow ektacytometer RheoScanAnD 300 (RheoMeditech, Seoul, Korea). A 6 μL blood sample was mixed with 600 μL of polyvinylpyrrolidone—PVP (RheoMeditech, 360 kDa, viscosity: 30 ± 2 mPa·s, osmotic pressure: 310 mOsm/kg, pH = 7.4). Before the measurements, the blood-PVP solution was heated for 15 min to 37 °C. Next, 500 μL of the solution was put into plastic disposable reservoirs supplied by the manufacturer (RheoMeditech, Seoul, Korea). The obtained results are presented as a relationship between the elongation index (EI) and indicated shear stress, ranging from 0.5 to 20.0 Pa. To calculate the EI value, the formula EI = (L − W)/(L + W) (L—the length and W—the width of the diffraction pattern formed by RBCs flowing through the microchannel in the reservoir illuminated with 633 nm laser light) was used. The RBCs’ deformability results are presented as the maximum EI (EImax) measured at the highest shear stress rate (20 Pa).

The complete blood count (mean corpuscular volume (MCV), mean corpuscular hemoglobin concentration (MCHC) mean corpuscular hemoglobin (MCH), red cell distribution width (RDW), hemoglobin (HGB), and hematocrit (HCT)) was performed for each sample using the hematology analyzer Abc Vet (Horiba Medical, Montpellier, France). Before, the measurement samples were mixed and tested according to manufacturer’s instructions.

The plasma removed after the first centrifugation of the whole blood was utilized for the biochemical analysis of glucose, creatinine, cholesterol, HDL, LDL, triglycerides, LDH, phosphorous, and iron. The levels of these parameters were assessed with use of a biochemical analyzer ABX Pentra 400 (Horiba Medical, Kyoto, Japan) using colorimetric methods. The necessary calibrations and control measurements were performed before the analysis. The measurements were carried out twice with the use of original reagents (Horiba ABX, Montpellier, France) and in accordance with the procedure delivered by the manufacturer.

Ektacytometry, hematologic, and biochemical data were analyzed with use of OriginPro software [62]. The results were presented as box diagrams or interval plots with median and max–min whiskers. Additionally, to identify statistically significant differences, the data were tested using a Mann–Whitney test.

### 4.4. Fourier Transform Infrared Spectroscopy–Attenuated Total Reflectance (FTIR–ATR) Measurements with Data Processing

For the FTIR–ATR studies, smears of isolated RBCs were prepared by applying 15 µL of the sample on CaF_2_ slides and air-drying for one hour. Samples of the isolated RBC membranes were prepared in an analogous manner, by depositing 15 µL of the samples on CaF_2_ slides.

Dried samples were scratched off with a spatula from CaF_2_ slides and placed on the ATR crystal. A Bruker Alpha FTIR spectrometer (Bruker Optics, MA, USA) equipped with a single reflection diamond ATR crystal was used to record the FTIR–ATR spectra. For each sample, three spectra were obtained with a spectral resolution of 4 cm^−1^, ranging from 3800 to 900 cm^−1^ by co-adding 512 scans. Then, on the received spectra, an ATR correction was made according to the implementation in OPUS software (Bruker Optics, version: 7.2.139.1294) [63]. Further processing of data was also performed in the OPUS software for the FTIR–ATR spectra. Afterwards, all the spectra were normalized in the range from 3600 to 900 cm^−1^. The second derivative of the spectra was calculated with use of the Savitzky–Golay method, with thirteen smoothing points. Integral intensities were calculated for the selected bands by determining the area under the peak. The obtained numerical values were presented in the form of box diagrams using OriginPro software (OriginLab, USA) [62]. Spectra were averaged for each sample (5 for RBC membranes and 15 for whole RBCs) and compiled together with their variations in Figure S3.

### 4.5. Raman Spectroscopy (RS) Measurements with Data Processing

Samples of isolated RBC membranes for RS studies were prepared by air-drying of the smears on CaF_2_ slides.

Measurements were performed using a WITec CRM alfa 300 confocal Raman microscope (WITec GmbH, Ulm, Germany) with an air-cooled semiconductor laser with a 488 nm wavelength and a CCD detector cooled to −60 °C. The laser was linked to the microscope with a 50 μm diameter optical fiber. The spectrometer’s monochromator was calibrated with the radiation spectrum of a xenon lamp (WITec UV light). An Olympus MPLAN dry objective (100/0.90NA) was used. The laser power at the sample position was about 10 mW. The acquisition time per spectrum was 3 s and the spectral resolution was 3 cm^−1^. Raman measurements and data analysis were performed applying WITec Project Plus 2.10 (WITec GmbH), OPUS (Bruker Optics), and OriginPro (OriginLab) software [62,64]. All averaged RS spectra were pre-processed (cosmic ray removal, smoothing—13 smoothing points, background removal) and normalized.

## 5. Conclusions

**Figure 6:**
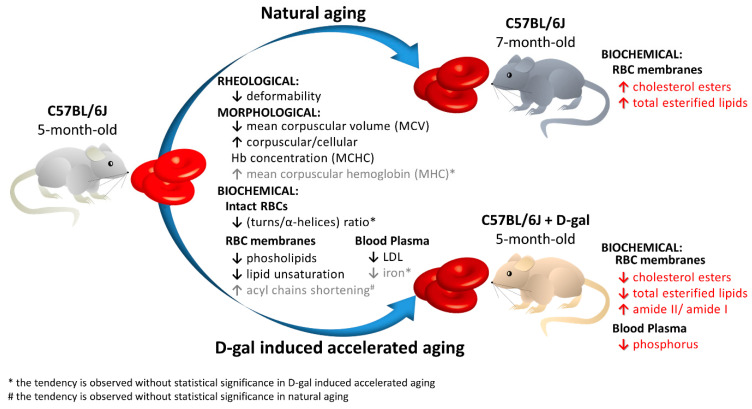
Schematic diagram of the RBC changes during natural aging compared with accelerated aging induced by D-galactose.

Based on the evidence provided in our work, summarized in Figure 6, the model of D-galactose-induced accelerated aging (D-gal) should be applied with caution for aging studies. The D-gal model mimics the mechanical and functional properties of RBCs in natural aging. The deterioration of RBC morphology includes a decrease in MCV and an increase in MCHC, the disturbance of the biochemical composition of the RBC membranes, a significant decrease in phospholipids and lipid unsaturation, and an increase in acyl chain shortening in the D-gal model compared to control animals. All the above-mentioned alterations, as well as those seen in the main blood plasma components follow the trends observed in the natural aging of C57BL/6J mice. However, senescent changes in the biochemical profile of RBC membranes were not identical for the D-galactose-induced accelerated aging model and natural aging model. Alterations in the secondary structures of proteins, as well as a decrease in the cholesterol esters and total esterified lipids of the RBC membranes were unique for the D-gal model, differentiating it from the natural aging of C57BL/6J mice.

## Data Availability

The data presented in this study are openly available in Jagiellonian University Repository at DOI: 10.26106/6kw9-k641.

## Supplementary Materials

Supplementary materials are available online at https://www.mdpi.com/1422-0067/22/5/2660/s1.
