# Supplementary material for: Spectroscopic Signature of Red Blood Cells in a D-Galactose-Induced Accelerated Aging Model
Source: Int J Mol Sci. 2021 Mar 6;22(5):2660. doi: 10.3390/ijms22052660 (PMC7961785; doi:10.3390/ijms22052660)
Supplement: Supplementary file 1 [file ijms-22-02660-s001.pdf]

International Journal of *Molecular Sciences*

Article

Spectroscopic Signature of Red Blood Cells in the D-galactose-Induced Accelerated Aging Model

Aneta Blat[1,2,†], Tetiana Stepanenko[1,2,†], Katarzyna Bulat[1], Aleksandra Wajda[1,3], Jakub Dybas[1], Tasnim Mohaissen[1], Fatih Celal Alcicek[1], Ewa Szczesny- Malysiak[1], Kamilla Malek[2], Andrzej Fedorowicz[4] and Katarzyna M. Marzec[1,*]

[1] Jagiellonian Center for Experimental Therapeutics, Jagiellonian University, 14 Bobrzynskiego Str., 30–348 Krakow, Poland; katarzyna.bulat@jcet.eu (K.B.); tasnim.mohaissen@jcet.eu (T.M.); f.celal.alcicek@jcet.eu (F.C.A.); jakub.dybas@jcet.eu (J.D.); ewa.szczesny@jcet.eu (E.S-M.)

[2] Faculty of Chemistry, Jagiellonian University, 2 Gronostajowa Str., 30–387 Krakow, Poland; aneta.blat@doctoral.uj.edu.pl (A.B.); tetiana.stepanenko@student.uj.edu.pl (T.S.); kamilla.malek@uj.edu.pl (K.M.)

[3] Faculty of Materials Science and Ceramics, AGH University of Science and Technology, Al. Mickiewicza 30, 30-059 Krakow, Poland; olawajda@agh.edu.pl (A.W.)

[4] Chair of Pharmacology, Jagiellonian University, 16 Grzegorzecka Str., 31-531 Krakow, Poland; andrzej.fedorowicz@uj.edu.pl (A.F.)

* Correspondence: katarzyna.marzec@jcet.eu; Tel.: +48-12-664-5476 (K.M.M.)

† Contributed equally

Academic Editor: Firstname Lastname; Received: date; Accepted: date; Published: date

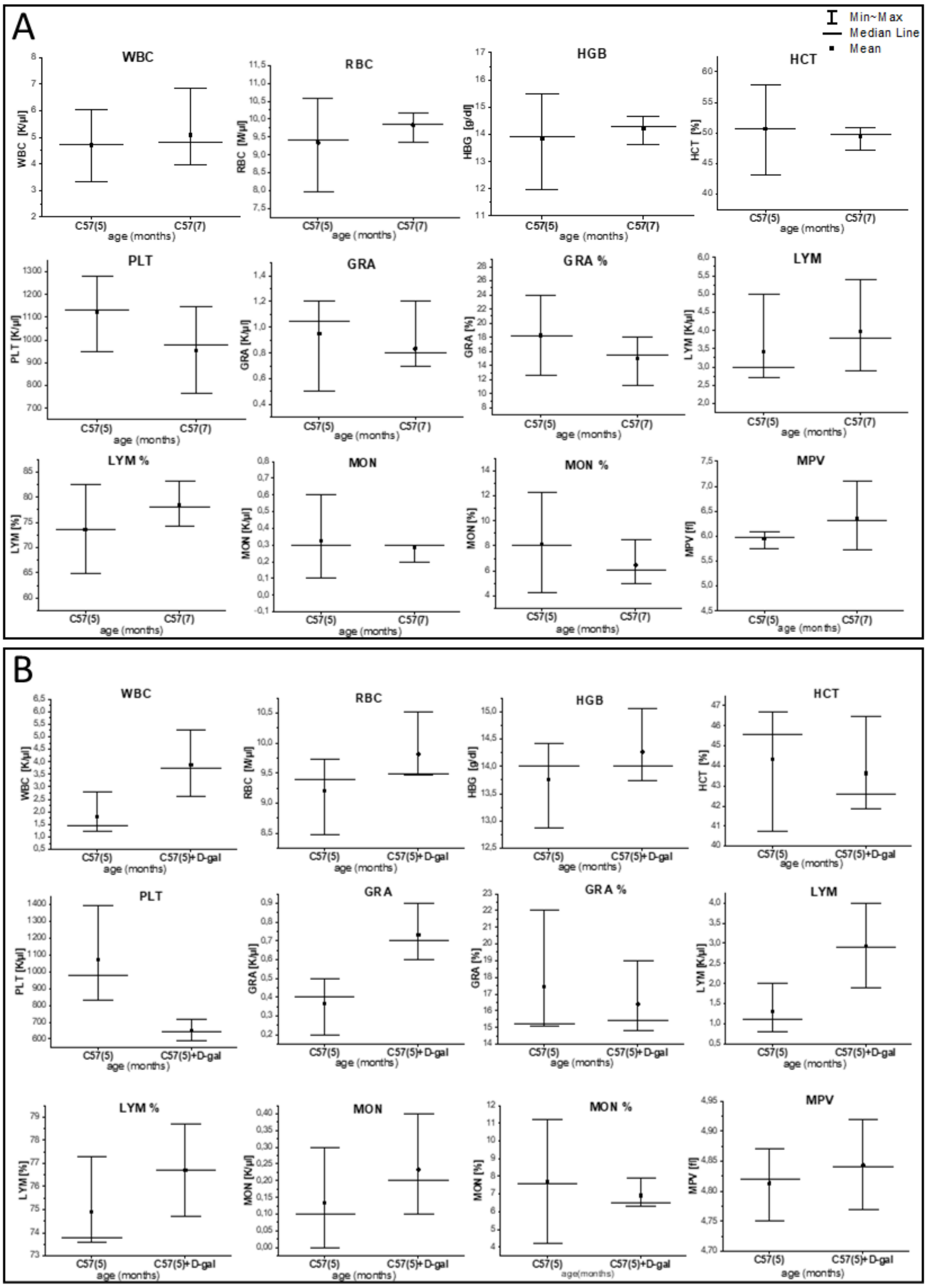

Figure S1. Changes in blood morphological parameters in natural aging and D-gal model. WBC, RBC, HGB, HCT, PLT, GRA, LYM, MON, MPV for (A) 5- and 7-month-old male C57BL/6J mice (N=4 and N=6, respectively) in natural aging and (B) 5-month-old male C57BL/6J mice in D-galactose induced aging model (N=3) with control group (N=3). Data distribution is presented as interval plots (mean value, median, min-max whiskers). Statistical significance of the obtained values was tested with Mann-Whitney test.

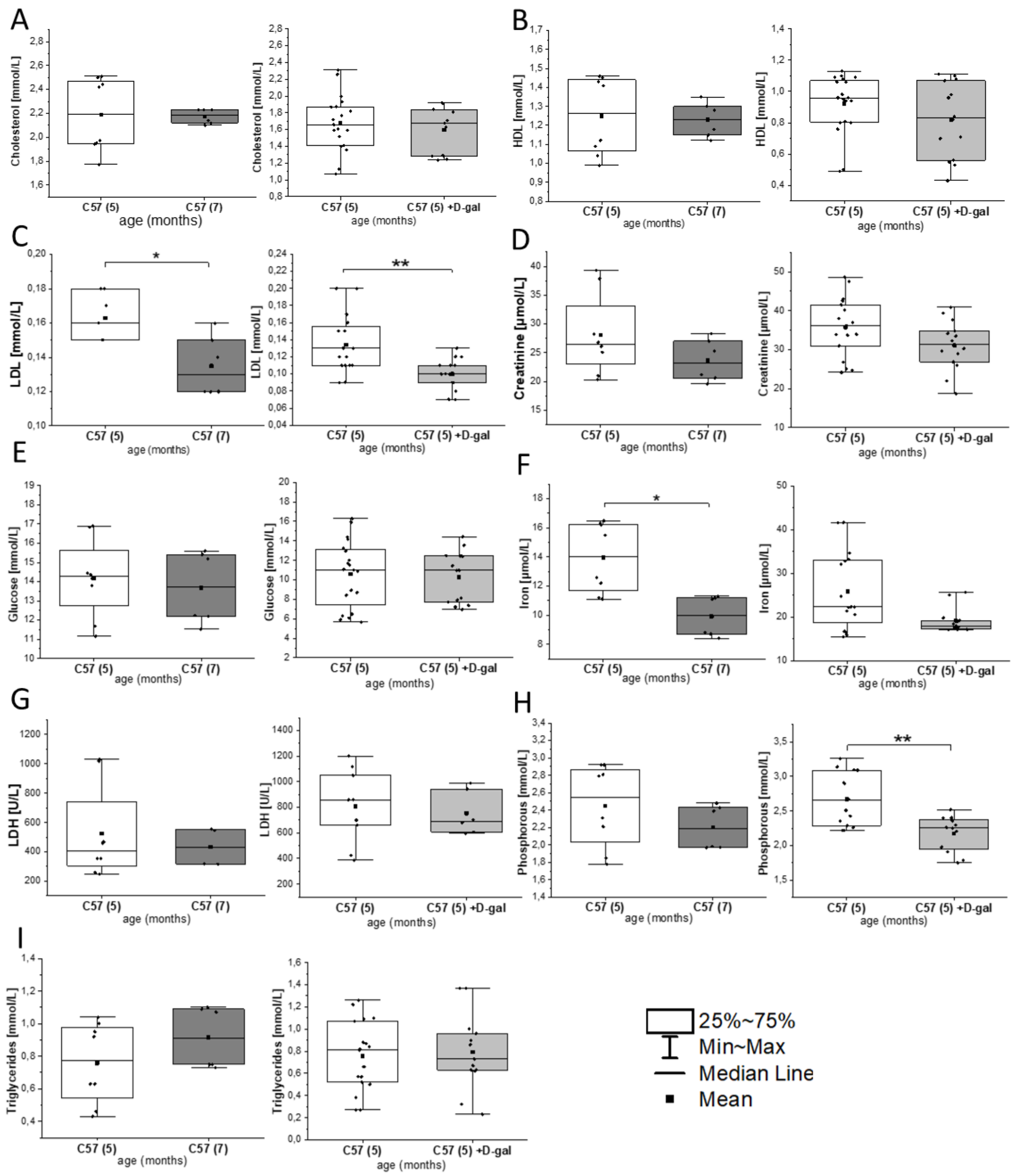

Figure S2. Biochemical parameters in blood plasma in natural aging and D-gal model. (A) Cholesterol, (B) HDL, (C) LDL, (D) creatinine, (E) glucose, (F) iron, (G) LDH, (H) phosphorous and (I) triglycerides level for 5- and 7-month-old male C57BL/6J mice (N=4 and N=3, respectively) in natural aging and 5-month-old male C57BL/6J mice in D-galactose induced aging model (N=7) with control group (N=10). Data distribution is presented as box (median and interquartile range, min-max whiskers). Statistical significance of the obtained values was tested with Mann-Whitney test (*p < 0.05; **p < 0.01).

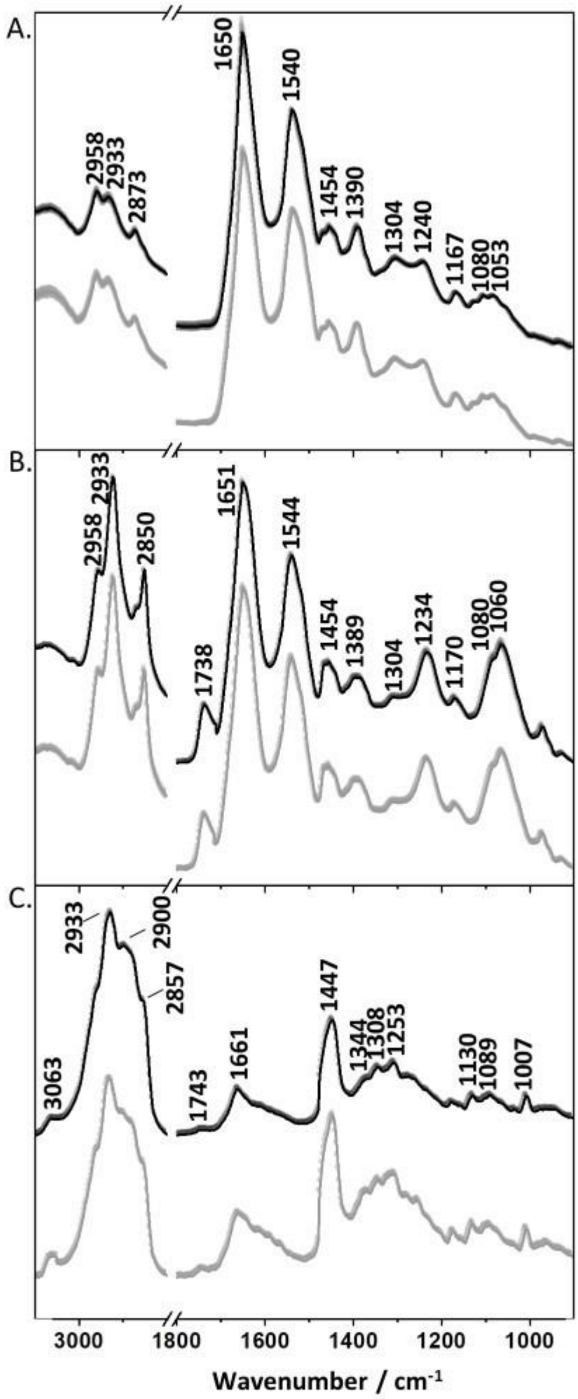

Figure S3. Averaged FTIR and Raman spectra with SD for RBCs and their membranes in D-gal model. FTIR spectra of intact RBCs (A) and isolated membranes (B) and Raman spectra of isolated membranes (C) taken from control (grey) and D-galactose induced accelerated aging mice (black).

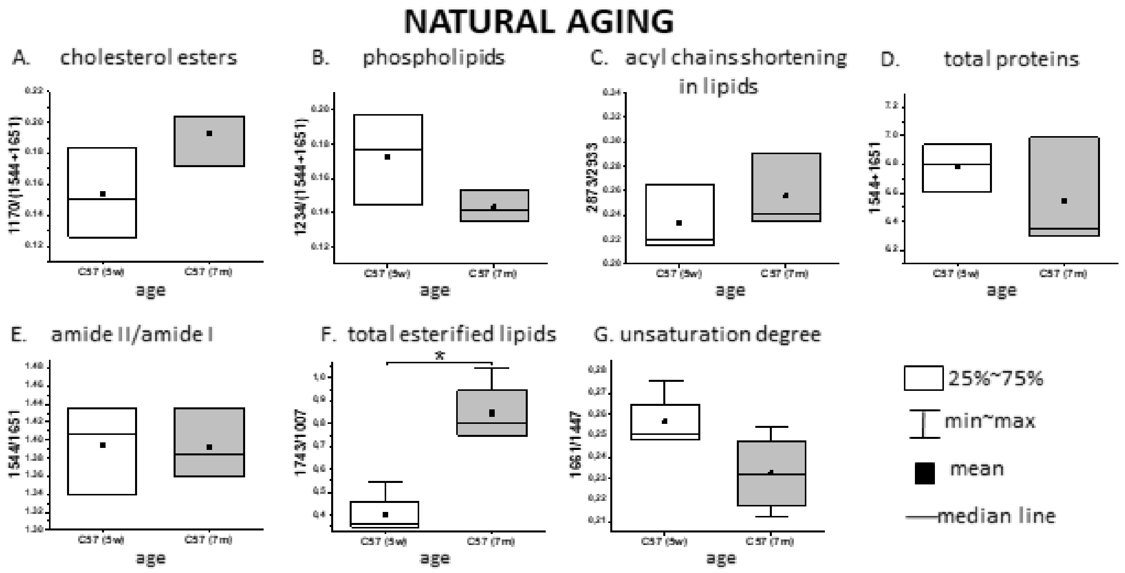

Figure S4. Spectroscopically – derived biochemical profile of RBC membranes in natural aging.

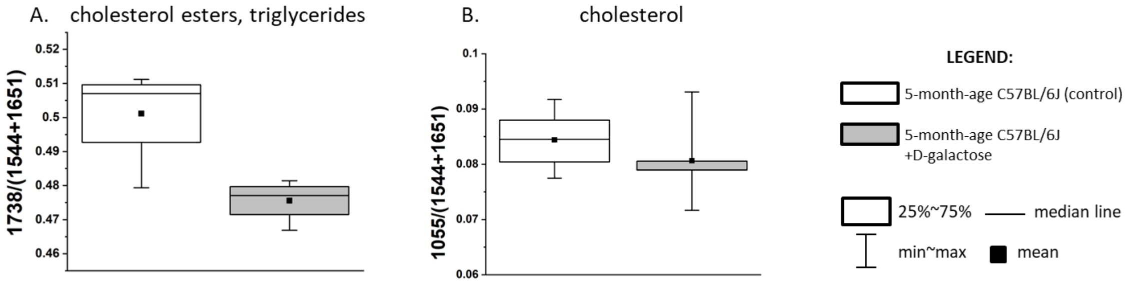

Figure S5. Alterations inbiochemical composition of RBC membranes due to the D-galactose induced accelerated aging (C57BL/6J and D-galactose-fed C57BL/6J mice). Ratios calculated for the integral absorbances in the ATR-FTIR spectra. **Integration regions for IR bands**: amide I – 1651 cm^{-1} (1687–1605 cm^{-1}); amide II – 1544 cm^{-1} (1560–1502 cm^{-1}); C=O stretch- 1738 cm^{-1} (1758-1727 cm^{-1}), 1055 cm^{-1} (1076-1034 cm^{-1}).

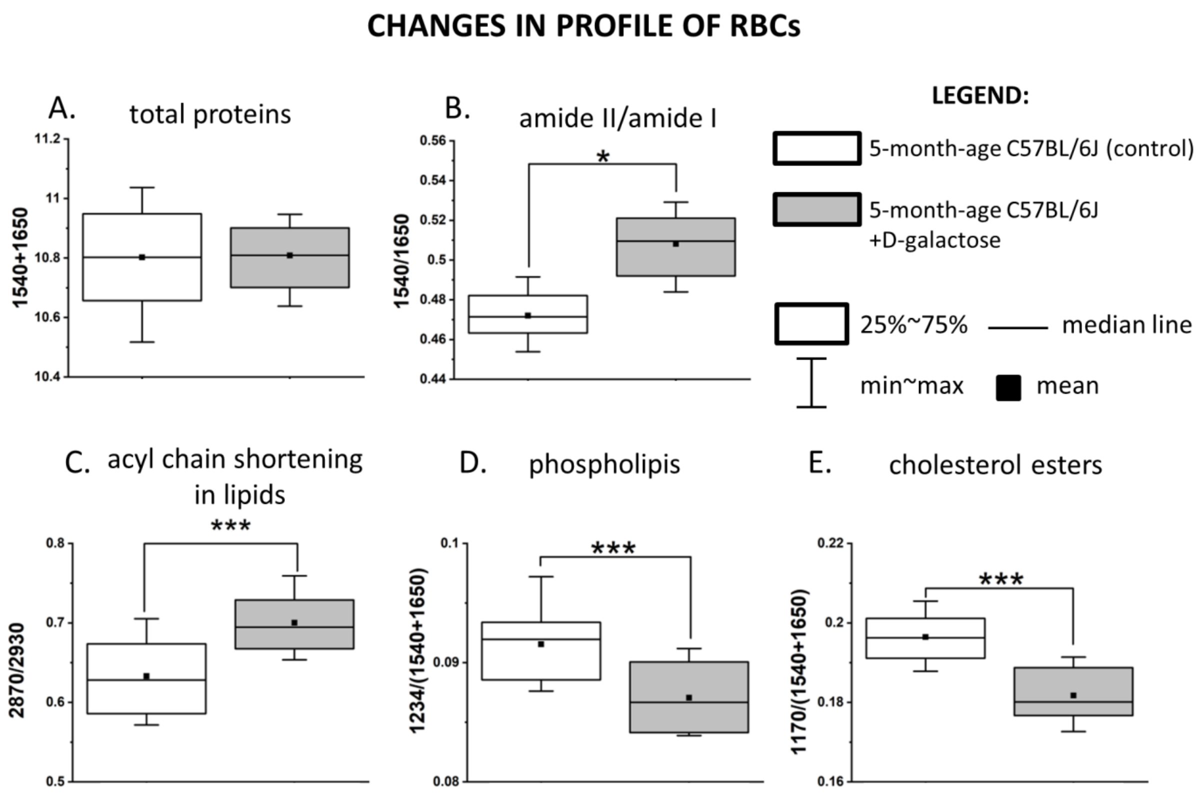

Figure S6. Alterations inbiochemical composition of intact RBCs due to the D-galactose-induced accelerated aging (C57BL/6J and D-galactose-fed C57BL/6J mice). Ratios calculated for the integral absorbances in the ATR-FTIR spectra. **Integration regions for IR bands**: amide I – 1651 cm^{-1} (1687–1605 cm^{-1}); amide II – 1544 cm^{-1} (1560–1502 cm^{-1}); CH_2 symmetric stretch – 2933 cm^{-1} (2863–2847 cm^{-1}); CH_3 asymmetric stretch – 2873 cm^{-1} (2965–2936 cm^{-1}); PO_2^- asymmetric stretch – 1236 cm^{-1} (1261–1214 cm^{-1}); –CO–O–C stretch – 1167 cm^{-1} (1191–1144 cm^{-1}).
